# Autophagy induction contributes to the resistance to methotrexate treatment in rheumatoid arthritis fibroblast-like synovial cells through high mobility group box chromosomal protein 1

**DOI:** 10.1186/s13075-015-0892-y

**Published:** 2015-12-23

**Authors:** Ke Xu, Yong-song Cai, She-Min Lu, Xiao-li Li, Lin Liu, Zhong Li, Hui Liu, Peng Xu

**Affiliations:** Department of Joint Surgery, Xi’an Hong Hui Hospital, Xi’an Jiaotong University Health Science Center, Xi’an, 710054 Shaanxi Province China; Department of Genetics and Molecular Biology, Xi’an Jiaotong University Health Science Center, Xi’an, Shaanxi Province China; Department of Internal Medicine, Xi’an Hong Hui Hospital, Xi’an Jiaotong University Health Science Center, Xi’an, Shaanxi Province China

**Keywords:** Rheumatoid arthritis, Fibroblast-like synovial cells, Methotrexate, Autophagy, Apoptosis

## Abstract

**Background:**

Rheumatoid arthritis fibroblast-like synovial cells (RA-FLS) show resistance to methotrexate (MTX) treatment. To better understand the mechanisms of this resistance, RA-FLS and osteoarthritis fibroblast-like synovial cells (OA-FLS) were isolated and exposed to MTX. We analyzed the autophagy induced by MTX in vitro and its relationship to apoptosis.

**Methods:**

Cell viability was evaluated using a 3-(4,5-dimethylthiazol-2-yl)-2,5-diphenyltetrazolium bromide assay, and apoptosis was detected by flow cytometry and Western blot analysis. Autophagy was determined by transmission electron microscopy as well as Western blot analysis. The expression levels of Beclin-1, LC3, Akt, p-Akt, mammalian target of rapamycin (mTOR), p-mTOR, high mobility group box chromosomal protein 1 (HMGB1), and an 85 kDa caspase cleaved fragment of poly(ADP-ribose) polymerase were measured by Western blotting.

**Results:**

MTX-induced apoptosis was increased in OA-FLS compared with RA-FLS. However, MTX stimulated the autophagy response in RA-FLS by inducing autophagosome formation, but not in OA-FLS. In RA-FLS, transfection with Beclin-1 small interfering RNA inhibited autophagy and increased susceptibility to MTX, which induces cell death. MTX upregulated autophagy through its ability to enhance the expression of HMGB1 and Beclin-1 rather than through the Akt/mTOR pathway.

**Conclusions:**

Autophagy induction contributes to resistance to MTX treatment in fibroblasts from patients with rheumatoid arthritis.

**Electronic supplementary material:**

The online version of this article (doi:10.1186/s13075-015-0892-y) contains supplementary material, which is available to authorized users.

## Background

With a high prevalence and an associated level of disability, rheumatoid arthritis (RA) is the most common serious autoimmune disease in several parts of the world. To date, it is widely accepted that both the inflammatory and destructive features of RA are driven by synovitis, characterized not only by increases in the number and activity of lymphocytes and macrophages but also by the numbers of resident mesenchymal cells, known as fibroblast-like synovial cells (FLS) [1]. Located in the synovial lining, FLS derived from RA appear to change their phenotype to become hyperplastic and invasive, like tumor cells [2]. Accumulating evidence indicates that the transformation of rheumatoid arthritis fibroblast-like synovial cells (RA-FLS) occurs as a result of both defective apoptosis and excessive proliferation.

The folic acid antagonist methotrexate (MTX), which is a potent, competitive inhibitor of dihydrofolate reductase, is the most widely used of the disease-modifying antirheumatic drugs (DMARDs) in the treatment of RA [3, 4]. Compared with the 5000 mg/week dosage used in the treatment of malignancy, once-weekly administration of MTX at 7.5–25 mg produces optimal clinical outcomes in RA [5]. According to previous studies, apart from the anti-inflammatory effects, MTX suppresses the proliferation of lymphocytes and macrophages but has no effect on spontaneous proliferation of RA-FLS [6, 7]. Its promotion of RA-FLS apoptosis is rather limited [8–11].

Autophagy is a survival strategy employed by cells undergoing nutrient deprivation or other stresses. Increasing evidence indicates that autophagy protects various tumor cells from apoptosis induced by chemotherapy drugs, both in vivo and in vitro [12–14]. Nevertheless, extensive or persistent autophagy also produces cell death. Thus, autophagy often serves as an adapter between cell death and survival [15]. Compared with osteoarthritis (OA), both enhanced autophagy in RA synovial tissues and increased induction of autophagy in RA-FLS were recently described [16, 17]. Autophagy exerted protective effects in these studies. This raises a question about the role of autophagy in the process of MTX treatment of RA-FLS.

On the basis of the present study, we report that MTX induced protective autophagy in RA-FLS and that inhibition of autophagy enhanced MTX-induced apoptosis of RA-FLS.

## Methods

### Human tissue collection

Synovial tissue specimens were obtained from seven patients with OA and seven patients with RA during joint replacement surgery (Department of Joint Surgery, Hong Hui Hospital, Xi’an, China). This study was approved by the human research ethics committee of the Xi’an Hong Hui Hospital, and all patients fulfilled the American College of Rheumatology criteria for classification of RA. All patients provided informed consent. Clinical data, laboratory examination results, and patient medications are summarized in Table 1.

**Table 1:** Patient characteristics

Patient characteristics	OA	RA
Total number of patients	7	7
Age,^a^ yr	68 (58–70)	61 (55–75)
Sex, *n*, female/male	2/5	5/2
CRP,^b^ mg/L	2.72 (2.25)	17.75 (26.52)
RF,^b^ IU/ml	9.16 (2.90)	102.31 (70.07)
Anti-CCP–positive, *n*	0/7	6/7
ESR,^b^ mm/h	12.43 (6.55)	39 (15.12)
Medications, number of patients		
NSAIDs	7	7
Steroids	3	1
TWP	0	3
MTX	0	4

*CRP* C-reactive protein, *ESR* erythrocyte sedimentation rate, *MTX* methotrexate, *RF* rheumatoid factor, *CCP* cyclic citrullinated peptide, *NSAID* nonsteroidal anti-inflammatory drug, *TWP Tripterygium wilfordii* polyglycoside

^a^Median (range)

^b^Mean (standard error of the mean)

### Reagents and antibodies

MTX and antibodies against microtubule-associated protein 1 light chain 3 (anti-LC3) were purchased from Sigma-Aldrich (St. Louis, MO, USA). Anti-β-actin antibodies were purchased from Biosen (Beijing, China). High mobility group box chromosomal protein 1 (HMGB1), anti-poly(ADP-ribose) polymerase (PARP), anti-phosphorylated mammalian target of rapamycin (anti-p-mTOR), and anti-mTOR antibodies were purchased from Abcam (Cambridge, UK). Anti-Beclin-1, anti-Akt, and anti-phosphorylated Akt (anti-phospho-Akt; Ser473) antibodies were purchased from Cell Signaling Technology (Danvers, MA, USA).

### Cell culture and treatment

Synovial fibroblasts were isolated from synovial tissue specimens obtained from patients with RA and patients with OA. Cells were cultured as described elsewhere [18] and used between passages 3 and 8 for all experiments. The concentrations of MTX used in the different experiments ranged from 0.01 μM to 1 μM, and the culture periods ranged from 24 h to 96 h of continuous exposure to MTX. On the basis of pharmacokinetic analysis, the ingestion of a 20-mg tablet of MTX yields plasma MTX concentrations of approximately 0.5 μM after 1 h and of approximately 0.1 μM after 10 h [19]. Controls were treated with matched amounts of dimethyl sulfoxide (DMSO); 0.1 μM of bafilomycin A1 (Sigma-Aldrich), which inhibits the fusion of autophagosomes with lysosomes, was added to cell cultures for the last 4 h of treatment.

### Cell viability assay

Cell viability was measured using a 3-(4,5-dimethylthiazol-2-yl)-2,5-diphenyltetrazolium bromide (MTT) assay. The cells were seeded at 5 × 10^4^ cells/well in 96-well plates, incubated overnight, and then exposed to the indicated concentrations of MTX for the indicated times. Thereafter, 20 μl of MTT solution (5 mg/ml) was added to each well, and the cells were incubated for another 4 h at 37 °C. After removal of the culture medium, the cells were lysed in 200 μl of DMSO, and the optical density (OD) was measured at 570 nm with a microplate reader (Thermo Fisher Scientific, Waltham, MA, USA). The following formula was used: cell viability = (OD of the experimental sample/OD of the control group) × 100 %.

### Analysis of cell death

After treatment, cells were detached with trypsin, washed twice with 1× phosphate-buffered saline (PBS), and resuspended in annexin V binding buffer (7SeaPharmTech, Shanghai, China) at a concentration of 3 × 10^5^ cells/ml. Next, the cells were incubated with fluorescein isothiocyanate–annexin V (7SeaPharmTech) for 15 minutes at room temperature in the dark and with propidium iodide (7SeaPharmTech) for 5 minutes at 4 °C in the dark, and then the cells were analyzed by flow cytometry (guava easyCyte HT; EMD Millipore, Billerica, MA, USA).

### Western blot analysis

Synovial fibroblasts and tissue specimens were washed twice with ice-cold PBS and solubilized in Triton X-100 lysis buffer (50 mM Tris–HCl, pH 7.4, 150 mM NaCl, 0.2 mM ethylenediaminetetraacetic acid, 1 % Triton X-100, 1 % sodium deoxycholate, 0.1 % sodium dodecyl sulfate [SDS]) and protease inhibitor cocktail (Beyotime, Shanghai, China) on ice, then quantified using the Lowry method. Cell lysate proteins (40 μg) were separated by SDS-polyacrylamide gel electrophoresis and electrophoretically transferred to nitrocellulose membranes (Immobilon-P; EMD Millipore). The membranes were blocked in 5 % skim milk in Tris-buffered saline with Tween 20 (TBST) at room temperature for 1 h and incubated overnight at 4 °C with the indicated primary antibodies. After a washing step with TBST buffer, the membranes were reacted with the appropriate horseradish peroxidase–conjugated secondary antibodies for 1 h at room temperature. After incubation with the secondary antibody, the membranes were washed three times with TBST and developed via electrochemiluminescence (Thermo Fisher Scientific) and using a Western blotting detection system (GeneGnome 5; Syngene, Cambridge, UK).

### Transmission electron microscopy

After treatment, cells were detached with trypsin, washed twice with PBS, and fixed in ice-cold 2 % glutaraldehyde/0.1 M phosphate buffer (pH 7.2), postfixed in 1 % osmium tetroxide, washed, dehydrated with a graded ethanol series (30 %, 50 %, 70 %, 90 %, and 100 %), and embedded in 1:1 propylene oxide/embedding resin. The resin blocks were cut with a LKB V ultramicrotome (LKB, Bromma, Sweden). Thin (60-nm) sections were picked up on 200-mesh copper grids and stained with uranyl acetate and lead citrate. The sections were examined with an H-7650 transmission electron microscope (HITACHI, Ibaraki, Japan).

### Transfection experiments

Both small interfering RNA (siRNA) targeting Beclin-1 complementary DNA (cDNA) sequence (5′-CAGTTTGGCACAATCAATATT-3′) and HMGB1 cDNA sequence (5′-CCCGTTATGAAAGAGAAATTT-3′) and a control siRNA (5′-UUCUCCGAACGUGUCACGUTT-3′) were obtained from Shanghai GenePharma (Shanghai, China). Cells were transfected with either Beclin-1 siRNA or control siRNA at 75 nmol/L using X-tremeGENE siRNA transfection reagent (Roche Diagnostics, Mannheim, Germany) according to the manufacturer’s guidelines. Twenty-four hours after transfection, the cells were treated as indicated and then harvested for Western blot analysis or flow cytometry.

### Statistical analysis

Mean ± SD values were calculated. According to whether data are normally distributed, either the Mann–Whitney *U* or Student’s *t* test was used for statistical evaluation of the data (GraphPad Prism 5.0 software; GraphPad Software, La Jolla, CA, USA). *p* Values less than 0.05 were considered significant.

## Results

### MTX inhibited cell viability and induced apoptosis

**Fig. 1:**
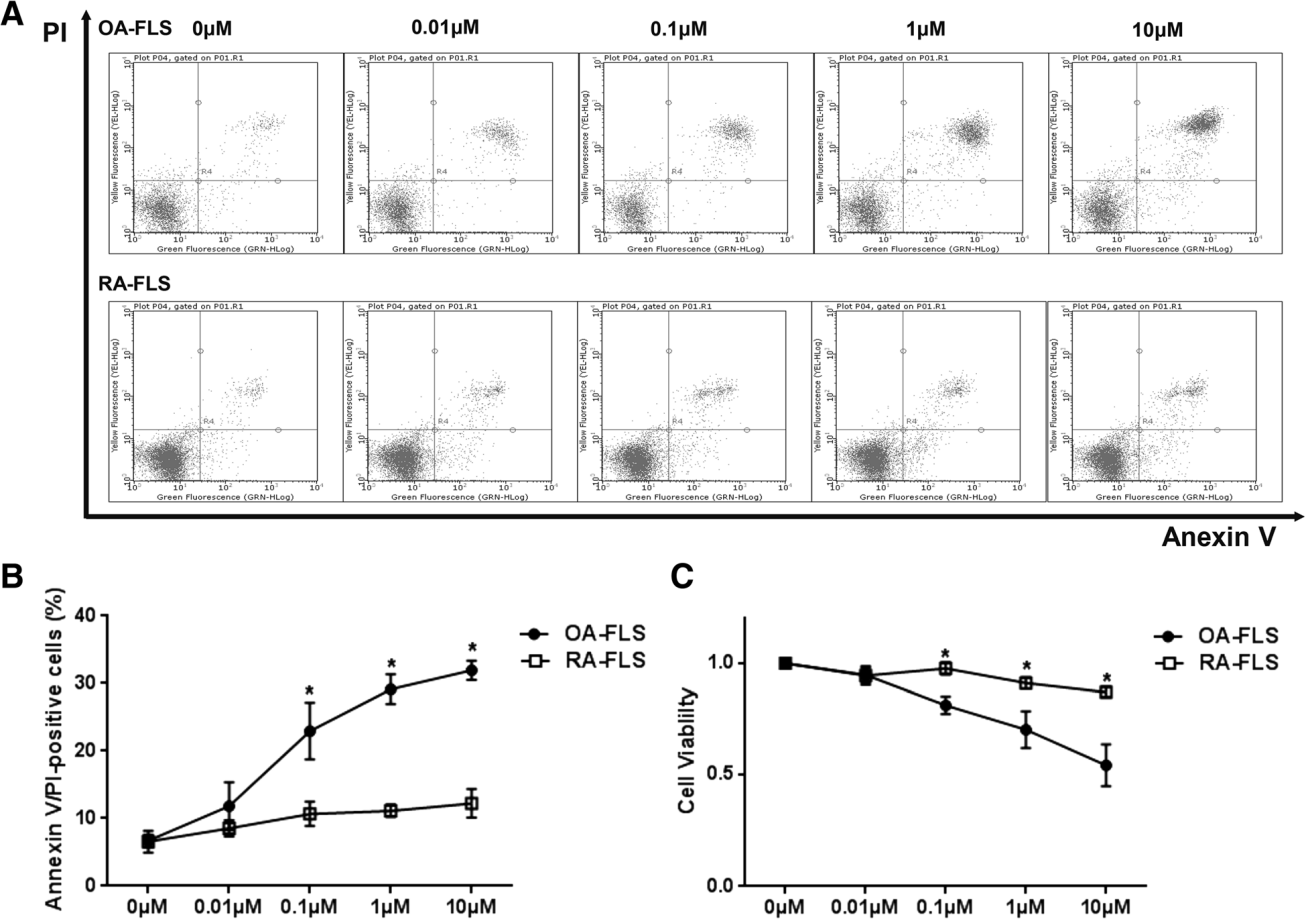
Effects of methotrexate (MTX) on cell viability and apoptosis of osteoarthritis fibroblast-like synovial cells (OA-FLS) and rheumatoid arthritis fibroblast-like synovial cells (RA-FLS). Cells were untreated or were treated with 0.01, 0.1, 1, or 10 μM MTX (**a** and **b**) for 48 h. The numbers of dead cells were determined by flow cytometry following annexin V/propidium iodide (PI) staining. Cells were treated with 0.01, 0.1, 1, or 10 μM MTX (**c**) for 48 h, and cell viability was analyzed by 3-(4,5-dimethylthiazol-2-yl)-2,5-diphenyltetrazolium bromide (MTT) assay. The results are representative of 4 experiments using cells from different patients. Values in (**b**) and (**c**) are the mean ± standard deviation. The Mann–Whitney *U* test was chosen for analysis of data from cell viability results of less than 0.01 μM MTX treatment in the MTT assay, while Student’s *t* test was used for the rest of the data. *p < 0.05 vs. OA-FLS

RA-FLS and osteoarthritis fibroblast-like synovial cells (OA-FLS) were treated with MTX at concentrations ranging from 0.01 μM to 10 μM for 48 h. By flow cytometry, assayed the number of dead cells after treatment with MTX, which showed that RA-FLS were more resistant than OA-FLS to MTX-induced cell death (Fig. 1a and b). Furthermore, cell viability assays showed that MTX inhibited cell growth in a dose-dependent manner (Fig. 1c). However, cell viability was 91.1 ± 2.5 % even when treated with MTX at a concentration of 1 μM in RA-FLS, in contrast to 70.2 ± 8.2 % in OA-FLS.

### MTX induced autophagosome formation

**Fig. 2:**
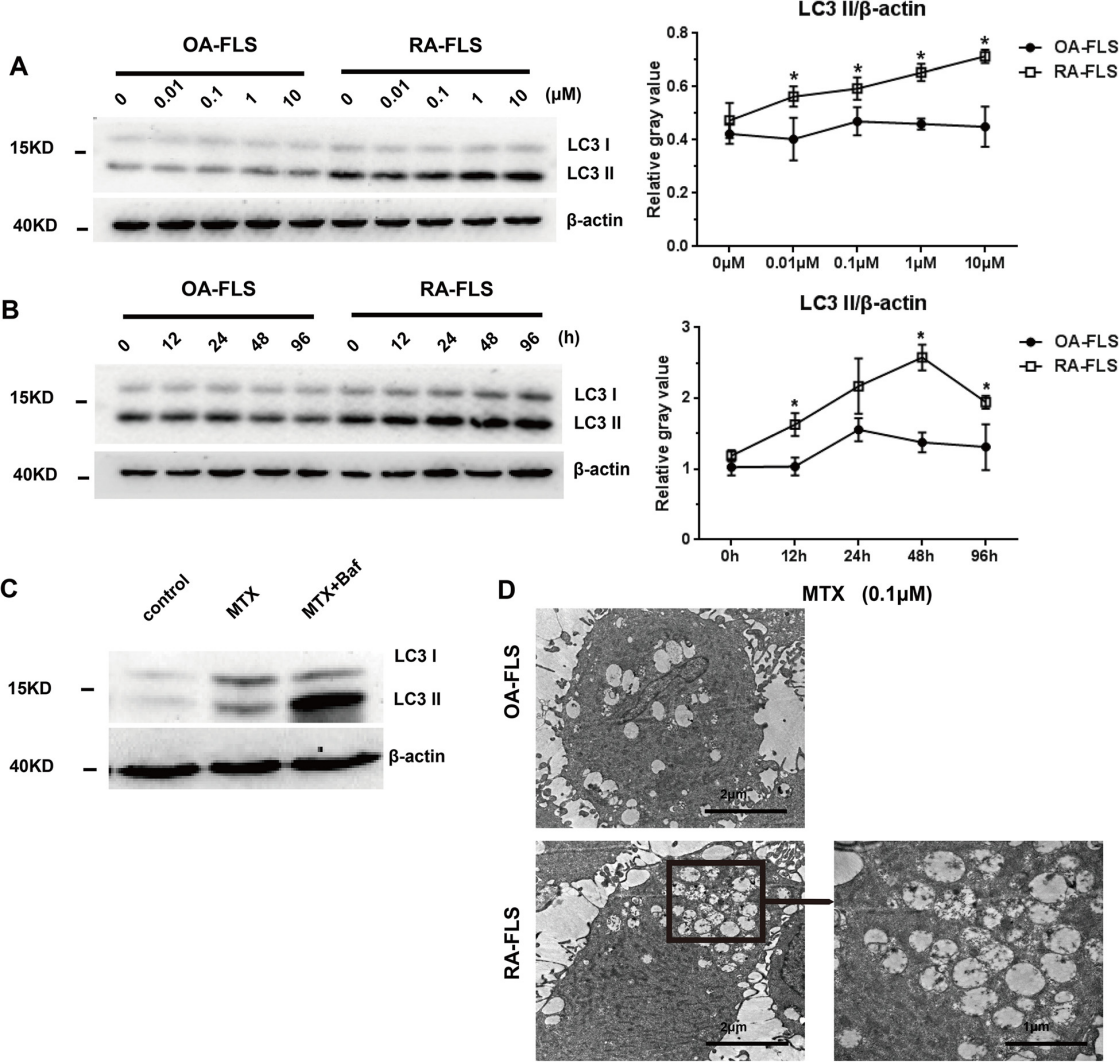
Determination of autophagy induction by monitoring the conversion of microtubule-associated protein 1 light chain 3 type I (LC3-I) to LC3-II in whole protein extracts derived from osteoarthritis fibroblast-like synovial cells (OA-FLS) and rheumatoid arthritis fibroblast-like synovial cells (RA-FLS). Cells were left untreated or were treated with 0.01, 0.1, 1, or 10 μM methotrexate (MTX) (**a**) for 48 h or 0.1 μM MTX (**b**) for the indicated times. Autophagic flux was monitored in RA-FLS after 24 h of treatment with 0.1 μM MTX in the presence or absence of 0.1 μM bafilomycin A1 (Baf) (**c**). Cells were treated with 0.1 μM MTX for 48 h (**d**), then harvested and subjected to transmission electron microscopy as described in the Methods section. The results are representative of three experiments using cells from different patients. β-actin was used as a loading control in all experiments. Values in (**a**) and (**b**) are the mean ± standard deviation. Student’s *t* test was used for the data analysis. *p < 0.05 vs. OA-FLS

Both RA-FLS and OA-FLS were treated with MTX at concentrations ranging from 0.01 μM to 10 μM for 48 h and at times ranging from 12 h to 96 h at a concentration of 0.1 μM. An increase in type II LC3 (LC3-II) was observed in both a dose-dependent a time-dependent manner after treatment with MTX (Fig. 2a and b), indicating the induction of autophagy. In addition, the induction of autophagy was more pronounced in RA-FLS than in OA-FLS (Fig. 2a and b).

The increase in LC3-II, which is indicative of autophagy induction, reflects only the number of autophagosomes formed; it provides no information about the overall autophagic flux. Therefore, in this study, we stimulated RA-FLS with MTX in the presence and absence of the lysosomal inhibitor bafilomycin A1 to block the autophagic pathway at a late stage [20]. We showed that MTX treatment also increased the autophagic flux in RA-FLS, as demonstrated by an increased amount of LC3-II in the presence of bafilomycin A1 (Fig. 2c).We also detected the expression of P62 to confirm the autophagic flux, but no significant changes were found (Additional file 1). The formation of autophagosomes was further confirmed by transmission electron microscopy. Upon treatment with 0.1 μM MTX for 48 h, many autophagic vesicles, double membrane–enclosed vesicles containing engulfed organelles, were observed in the cytoplasm of RA-FLS (Fig. 2d).

### MTX-induced autophagy protected RA-FLS from undergoing apoptosis

**Fig. 3:**
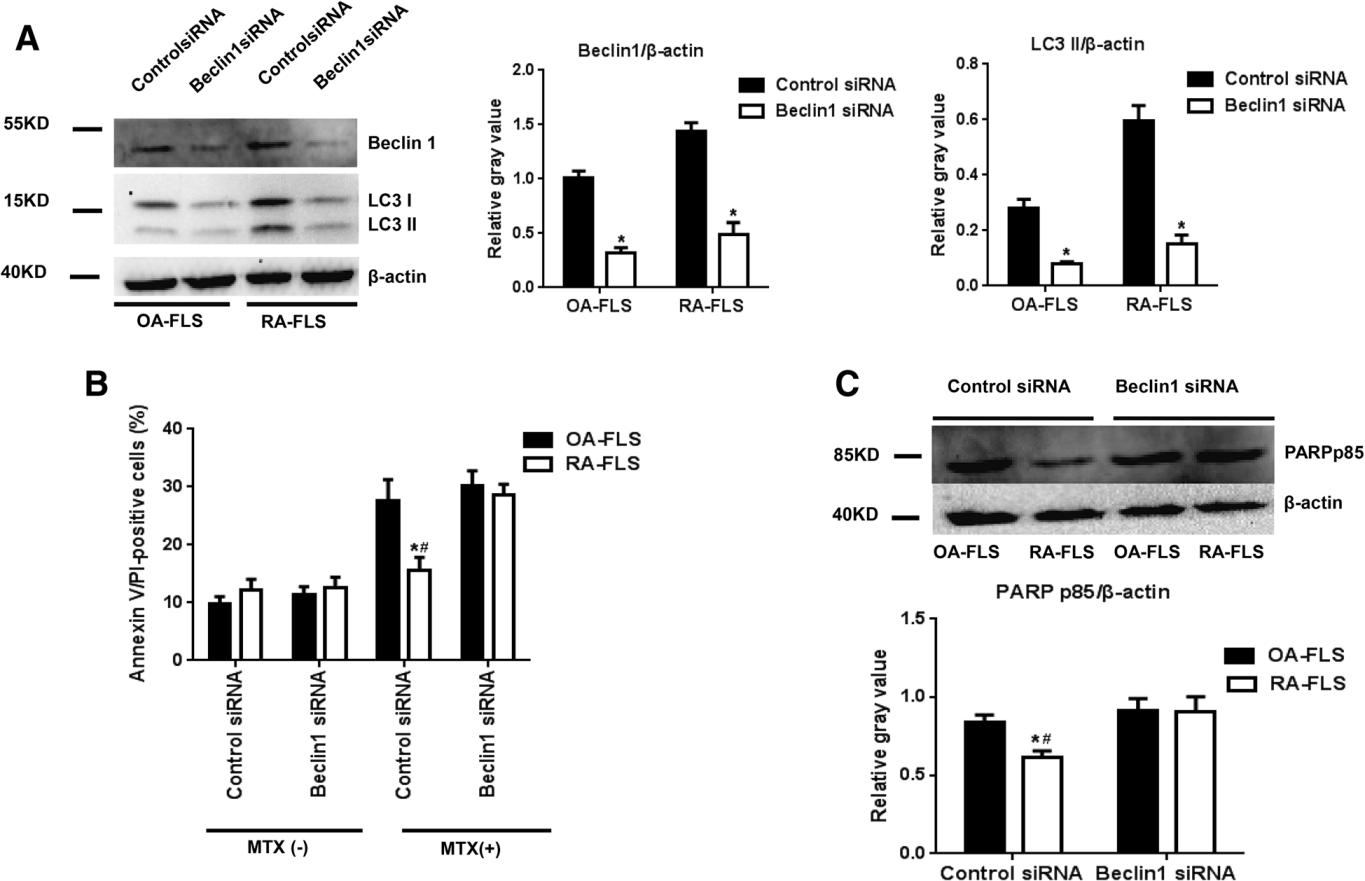
Inhibition of autophagy enhanced the proapoptotic effect of methotrexate (MTX) on osteoarthritis fibroblast-like synovial cells (OA-FLS) and rheumatoid arthritis fibroblast-like synovial cells (RA-FLS). Cells were transfected with control or Beclin-1 small interfering RNA (siRNA), followed by 0.1 μM MTX treatment for 48 h. The expression of Beclin-1 and microtubule-associated protein 1 light chain 3 types I and II (LC3-I and LC3-II, respectively) was verified by Western blot analysis (**a**). The numbers of dead cells were determined by flow cytometry following annexin V/propidium iodide (PI) staining (**b**). Verification of poly(ADP-ribose) polymerase (PARP) p85 protein expression by Western blot analysis (**c**). The results are representative of four experiments using cells from different patients. β-actin was used as a loading control in all experiments. Student’s *t* test was used for the data analysis. Values in (**a**) are the mean ± standard deviation (SD). *p < 0.05 vs. control siRNA. Values in (**b**) and (**c**) are the mean ± SD. *p < 0.05 vs. OA-FLS transfected with control siRNA. ^**#**^ *p* < 0.05 vs. RA-FLS transfected with Beclin-1 siRNA

Because autophagy can result in both survival and cell death in RA-FLS and OA-FLS, we next investigated whether MTX-induced autophagy is protective or proapoptotic. In this experiment, we examined the role of MTX-induced autophagy via knockdown of the autophagy marker Beclin-1. Figure 3a shows that the levels of Beclin-1 and LC3II were significantly decreased in Beclin-1 siRNA-treated cells compared with the results in siRNA controls. In RA-FLS, but not in OA-FLS, the Beclin-1 siRNA significantly increased the apoptotic population with 0.1 μM MTX (Fig. 3b). To further confirm that apoptosis was induced by MTX, Western blotting was performed to detect the cleavage of PARP. As shown in Fig. 3c, after 0.1 μM MTX treatment for 48 h, the cleavage of PARP was increased more dramatically in the RA-FLS transferred with Beclin-1 siRNA than with the control siRNA, but it showed no comparable change in OA-FLS transferred with Beclin-1 siRNA.

### MTX induced autophagy through HMGB1 and Beclin-1, not the Akt/mTOR pathway

**Fig. 4:**
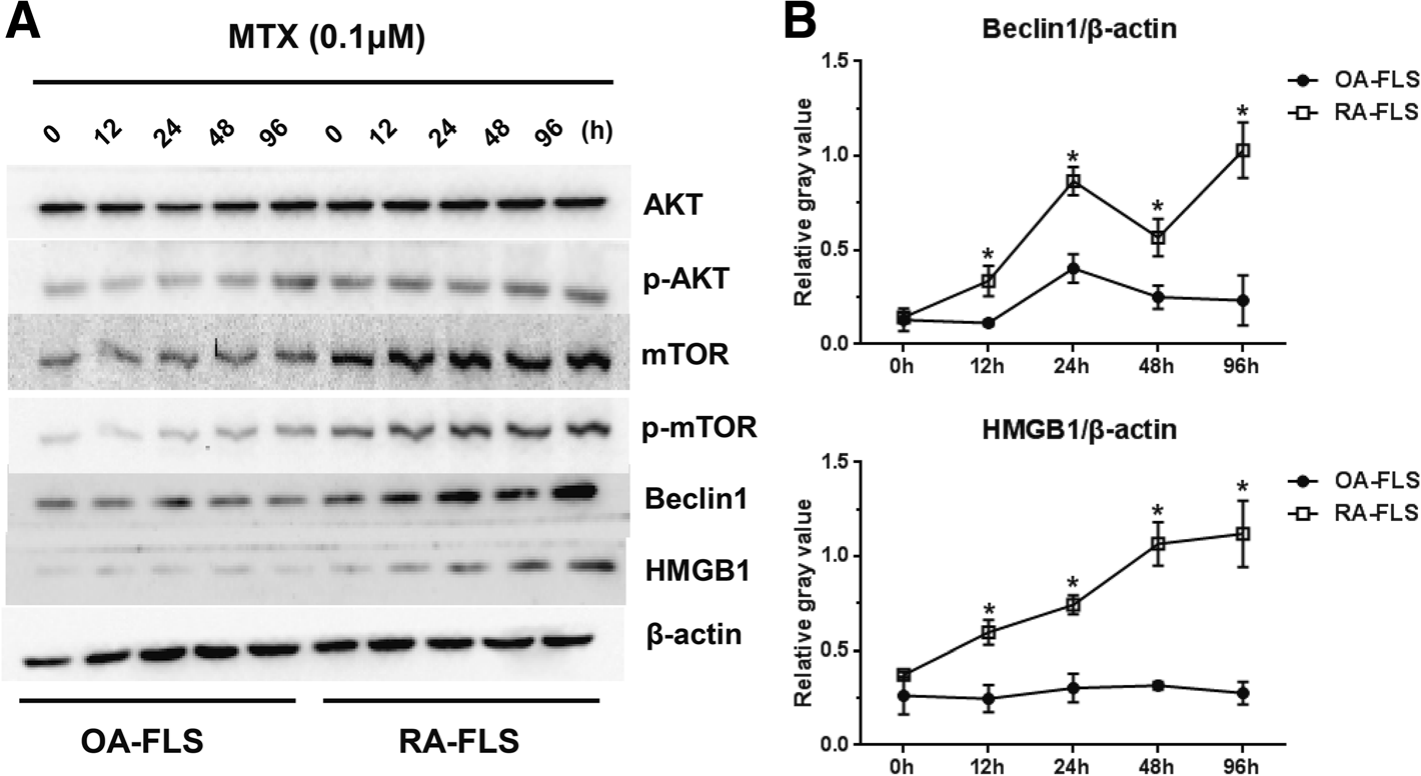
Effect of methotrexate (MTX) on autophagy-related proteins. **a** After the cells were exposed to 0.1 μM MTX for the indicated times, the cell lysates were subjected to Western blotting with specific antibodies. The results are representative of three independent experiments. β-actin was used as a loading control. **b** The levels of Akt, phosphorylated Akt (p-Akt), mammalian target of rapamycin (mTOR), phosphorylated mTOR (p-mTOR), high mobility group box chromosomal protein 1 (HMGB1), Beclin-1, and microtubule-associated protein 1 light chain 3 (LC3) proteins were measured using ImageJ software (National Institutes of Health, Bethesda, MD, USA) and corrected for β-actin. Data in columns represent mean of three independent experiments, and values in (**b**) are the mean ± standard deviation. The Mann–Whitney *U* test was chosen for analysis of data for the levels of HMGB1 of cells exposed to 0.1 μM MTX for 48 h in Western blot assays, while Student’s *t* test was used for the rest of the data. *p < 0.05 vs. OA-FLS

To explore how MTX induces autophagy in RA-FLS and OA-FLS treated with MTX at times ranging from 12 h to 96 h at a concentration of 0.1 μM, two important autophagy-related signaling pathways were investigated: the Akt/mTOR signaling pathway and the HMGB1/Beclin-1 pathway. As detected by Western blotting, the levels of phospho-Akt and downstream p-mTOR were more powerful in RA-FLS but were not affected by prolonging the duration of MTX in both RA-FLS and OA-FLS. Interestingly, the expression of HMGB1 and Beclin-1 was upregulated in a time-dependent manner in RA-FLS, but in OA-FLS it was very limited (Fig. 4a and b). These data indicated that MTX-induced autophagy might occur via upregulated HMGB1 and Beclin-1 rather than through the Akt/mTOR signaling pathway in RA-FLS.

### Autophagy induction contributed to resistance to MTX treatment in RA-FLS through HMGB1

**Fig. 5:**
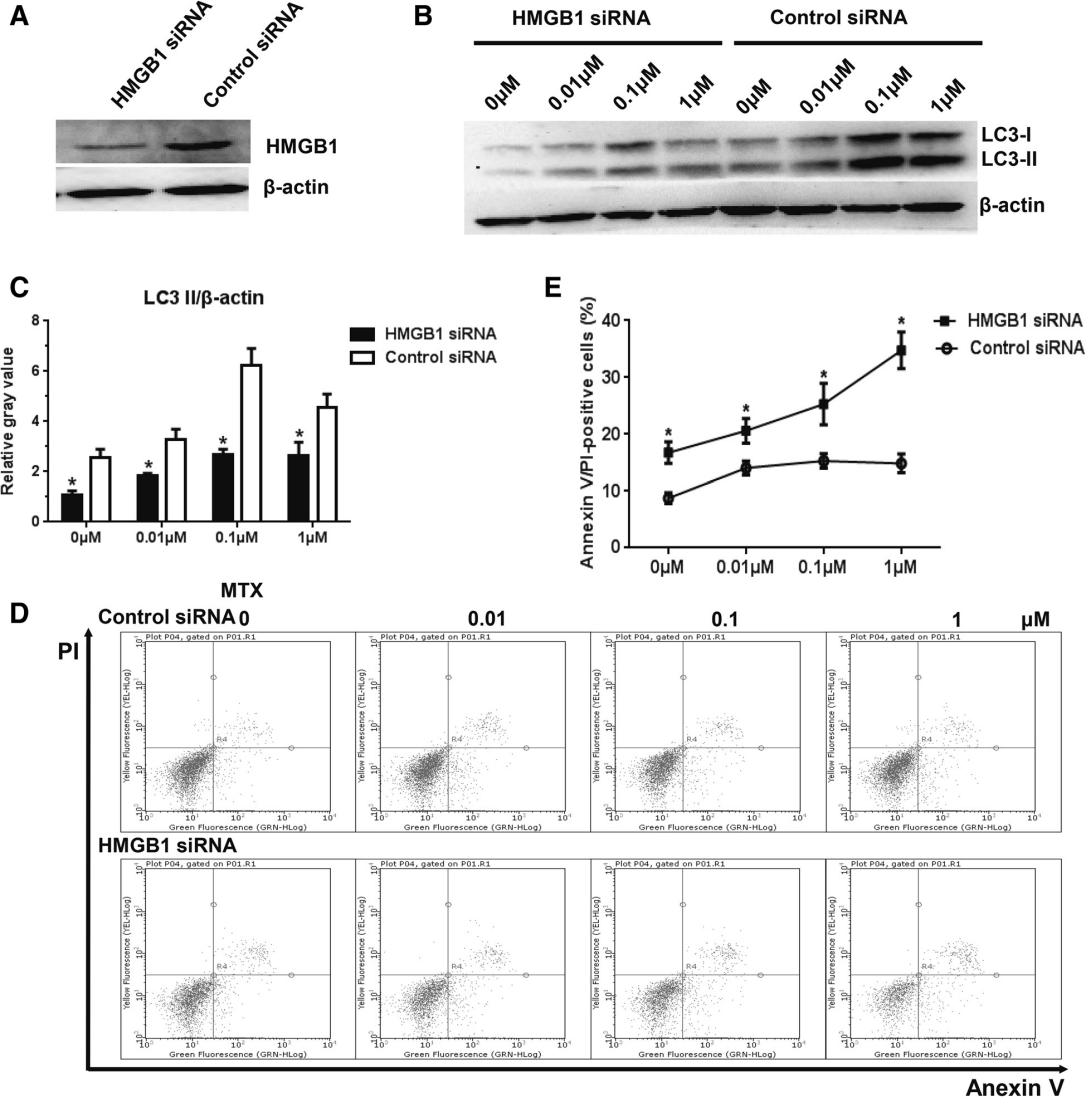
Autophagy induction contributed to the resistance to methotrexate (MTX) treatment in rheumatoid arthritis fibroblast-like synovial cells (RA-FLS) through high mobility group box chromosomal protein 1 (HMGB1). Cells were transfected with either HMGB1 small interfering RNA (siRNA) or a nontarget control siRNA for 48 h, and the expression of microtubule-associated protein 1 light chain 3, types I and II (LC3 I and LC3 II, respectively) was verified by Western blotting (**a**, **b**, and **c**). The numbers of dead cells were determined by flow cytometry following annexin V/propidium iodide (PI) staining (**d** and **e**). Data in columns represent the mean of three independent experiments, and values in (**c**) and (**e**) are the mean ± standard deviation. The Mann–Whitney *U* test was chosen for analysis of data for the numbers of dead cells without MTX treatment, while Student’s *t* test was used for the rest of the data. *p < 0.05 vs. control siRNA

We further confirmed the role of HMGB1 in MTX-induced autophagy via knockdown of the expression of HMGB1. Figure 5a–c shows that the protein levels of HMGB1 and LC3-II were significantly decreased in HMGB1 siRNA-treated cells compared with the results with the siRNA controls. In RA-FLS, the HMGB1 siRNA significantly increased the apoptotic population compared with the siRNA controls (Fig. 5d and e).

## Discussion

A substantial increase in the number of RA-FLS, partly but importantly resulting in synovial hyperplasia, is partially caused by resistant apoptosis. Different from either nonarthritic controls or patients with OA, RA-FLS hardly show any signs of apoptosis, which was also observed in animal model studies. Furthermore, RA-FLS also exhibited resistance to apoptotic stimulation in a number of in vitro studies. However, proliferation of RA-FLS seems to contribute to synovial hyperplasia. It has been reported in several research articles that some cellular proliferation regulators, such as Ras and c-Myc, are overexpressed in RA and that the inhibition of these regulators can reduce the growth of RA-FLS both in vitro and in vivo [21, 22].

One of the unique effects of MTX is the induction of RA-FLS apoptosis, compared with other DMARDs, which was first described by Nakazawa et al. [10]. Nevertheless, this effect was later shown to be compromised. Terminal deoxynucleotidyl transferase deoxyuridine triphosphate nick end labeling– and PARP-positive cells were found mainly in the synovial lining macrophages, but not in RA-FLS or other cell types, from RA synovial biopsy specimens after MTX treatment [11]. Lee et al. demonstrated apoptosis of RA-FLS treated with 100–500 μM MTX in cell culture [9], which is a concentration that cannot be achieved in the plasma. In the present study, we demonstrated a dose–effect relationship between MTX and apoptosis of RA-FLS and OA-FLS. However, apoptotic cells were detectable in the RA-FLS (11.1 ± 0.9 %) cultured with MTX at a high dose of up to 1 μM, significantly less than OA-FLS (29.2 ± 2.2 %). In contrast, pharmacokinetic analysis indicates that the ingestion of a 20-mg tablet of MTX yields plasma MTX concentrations of approximately 0.5 μM after 1 h and approximately 0.1 μM after 10 h.

Apart from the capacity of RA-FLS for apoptotic resistance, advanced sensitivity to induced autophagy seems to be another characteristic of RA-FLS that is in contrast to OA-FLS [11, 16, 17, 23]; however, it is rarely reported whether MTX has the ability to induce autophagy in RA-FLS. In our study, the increased expression of punctate LC3 and the morphologic changes were observed among the cells treated with MTX. Western blot analysis also showed that LC3-II expression was elevated with MTX treatment in a dose-dependent and time-dependent manner. If the increase in both LC3-II and autophagosome occurs only as a result of either upregulation of autophagosome formation or blockage of autophagic degradation, autophagic flux would be detected [24]. We found that in RA-FLS treated with 0.1 μM MTX, LC3-II further accumulated in the presence of bafilomycin A1, a lysosomal protease inhibitor. This finding indicates enhancement of autophagic flux and upregulated autophagosome formation by MTX [20]. To our knowledge, this is the first report that MTX induces autophagy in RA-FLS.

Autophagy has been shown to engage in a complex interplay with cell survival. Sometimes it induces an apoptotic or autophagic cell death accompanied by massive cytoplasmic vacuolization; at other times, it serves as a protector, as observed in RA-FLS. The studies with patients with RA by Shin et al. demonstrated that autophagy protects cells from death by limiting the endoplasmic reticulum stress response in fibroblasts [16]. However, a more recent study indicated a different result. Autophagy induced by different stimuli may lead to different and even opposite consequences. Autophagy seems to play a dual role in the survival of RA-FLS [23]. To clarify the consequences of autophagy induced by MTX, we inhibited autophagic activity by transferring Beclin-1 siRNA in RA-FLS, which significantly decreased the Beclin-1 expression and subsequently resulted in increased apoptosis with MTX treatment. Under our experimental conditions, MTX-induced autophagy served as a protector in RA-FLS.

The mTOR-dependent pathway is a classical regulator of autophagy [25]. The target of rapamycin (TOR) kinase is activated downstream of Akt kinase, phosphoinositide 3-kinase (PI3K), and growth factor receptor, signaling when nutrients are available. Upstream of TOR, the activation of adenosine 5′-monophosphate–activated protein kinase in response to low ATP levels promotes inhibitory activity of the Tsc1/Tsc2 tumor suppressor proteins on Rheb, a small GTPase required for mTOR activity. Reduced Akt activity in response to reduced growth factor receptor activity also represses TOR kinase through Tsc1 and Tsc2 [26, 27]. Thus, reduced TOR activity induces autophagy. In this study, the expressions of p-Akt and p-mTOR were higher in RA-FLS than in OA-FLS, but these seemed irrelevant to the use of MTX.

Autophagy induced under pathological conditions functions as an adaptive cell response that allows the cell to survive bioenergetic stress [28]. However, extensive or persistent autophagy also results in cell death [29]. Bielen et al. found that phosphorylation of platelet-derived growth factor receptor (PDGFR) α/β suppresses autophagy by activating the PI3K/Akt/mTOR signaling pathway, thereby preventing RA-FLS from undergoing type II apoptosis induced by excessive autophagy and leading to continuing proliferation of RA-FLS and aggravation of the pathogenetic condition [30]. The activation of the PI3K/Akt/mTOR signaling pathway may prevent RA-FLS from excessive autophagy and avoid type II apoptosis. Therefore, this may be another survival mechanism of drug treatment resistance. Nevertheless, why autophagy is robust in RA-FLS after MTX treatment remains unknown.

Fortunately, MTX induced in a time-dependent manner the expression of Beclin-1 and HMGB1 in RA-FLS, but this was very limited in OA-FLS. Beclin-1, the mammalian homolog of the yeast Atg6, is a key autophagy-promoting gene whose product is part of a lipid kinase (class III PI3K [PtdIns3KC3]) complex that participates in the early stages of autophagosome formation [31]. Upregulated HMGB1 competes with Bcl-2 to bind Beclin-1, which increases the formation of the Beclin-1–PtdIns3KC3 complex and stimulates autophagosome maturation and autophagy. As an upstream signal, the activation of the ULK1–mAtg13–FIP200 complex is required for the interaction between HMGB1 and Beclin-1 [32, 33]. We also demonstrated that autophagy activity was downregulated after transferring HMGB1 siRNA. Therefore, the robust autophagy of RA-FLS with MTX treatment seems to be caused by HMGB1.

In addition, we found that the inhibition of HMGB1 increased the apoptotic population of RA-FLS after MTX treatment. HMGB1 is an inducer of autophagy. Our study showed that knockdown of HMGB1 decreased LC3-II levels and inhibited autophagy activity, resulting in increased apoptosis, which is the same result found in non–small cell lung cancer [34]. Apart from its proautophagic effect and its interactions with receptor for advanced glycation endproducts (RAGE) and Toll-like receptor 4, HMGB1 plays a crucial role in various inflammation processes [35]. The induction of HMGB1 may mediate proinflammatory action in RA-FLS, while inhibition of the HMGB1–RAGE interaction may have anti-inflammatory effects in RA [36, 37]. The inhibition of the proinflammatory effects through the transfer of HMGB1 siRNA may be another cause of the increased drug sensitivity of RA-FLS. However, further study is required to explain how MTX induces the expression of HMGB1 and Beclin-1.

## Conclusions

The results of the present study suggest that RA-FLS may use the autophagic pathway in which HMGB1 and Beclin-1 (but not the Akt/mTOR pathway) are involved as a survival mechanism to evade the perturbations of cellular biosynthetic processes by the antimetabolite MTX to sustain cell viability in conditions of metabolic stress. Thus, a combination of MTX and an autophagy inhibitor might be more effective for the treatment of RA.

## Additional file

Additional file 1: The expression of P62 in RA-FLS and OA-FLS treated with 0.1μM MTX for the indicated times (TIF 698 kb)

## Abbreviations

Baf: bafilomycin A1
CCP: cyclic citrullinated peptide
cDNA: complementary DNA
CRP: C-reactive protein
DMARD: disease-modifying antirheumatic drug
DMSO: dimethyl sulfoxide
ESR: erythrocyte sedimentation rate
FLS: fibroblast-like synovial cells
HMGB1: high mobility group box chromosomal protein 1
LC3: microtubule-associated protein 1 light chain 3
LC3-II: microtubule-associated protein 1 light chain 3, type II
mTOR: mammalian target of rapamycin
MTT: 3-(4,5-dimethylthiazol-2-yl)-2,5-diphenyltetrazolium bromide
MTX: methotrexate
NSAID: nonsteroidal anti-inflammatory drug
OA: osteoarthritis
OA-FLS: osteoarthritis fibroblast-like synovial cells
OD: optical density
PBS: phosphate-buffered saline
PARP: poly(ADP-ribose) polymerase
PI: propidium iodide
PI3K: phosphoinositide 3-kinase
p-mTOR: phosphorylated mammalian target of rapamycin
PtdIns3KC3: class III phosphoinositide 3-kinase
RA: rheumatoid arthritis
RA-FLS: rheumatoid arthritis fibroblast-like synovial cells
RAGE: receptor for advanced glycation endproducts
RF: rheumatoid factor
SD: standard deviation
SDS: sodium dodecyl sulfate
siRNA: small interfering RNA
TBST: Tris-buffered saline with Tween 20
TOR: target of rapamycin
TWP: *Tripterygium wilfordii* polyglycoside
