# Supplementary figures and images for: Autophagy induction contributes to the resistance to methotrexate treatment in rheumatoid arthritis fibroblast-like synovial cells through high mobility group box chromosomal protein 1
Source: Arthritis Res Ther. 2015 Dec 23;17:374. doi: 10.1186/s13075-015-0892-y (PMC4718027; doi:10.1186/s13075-015-0892-y)

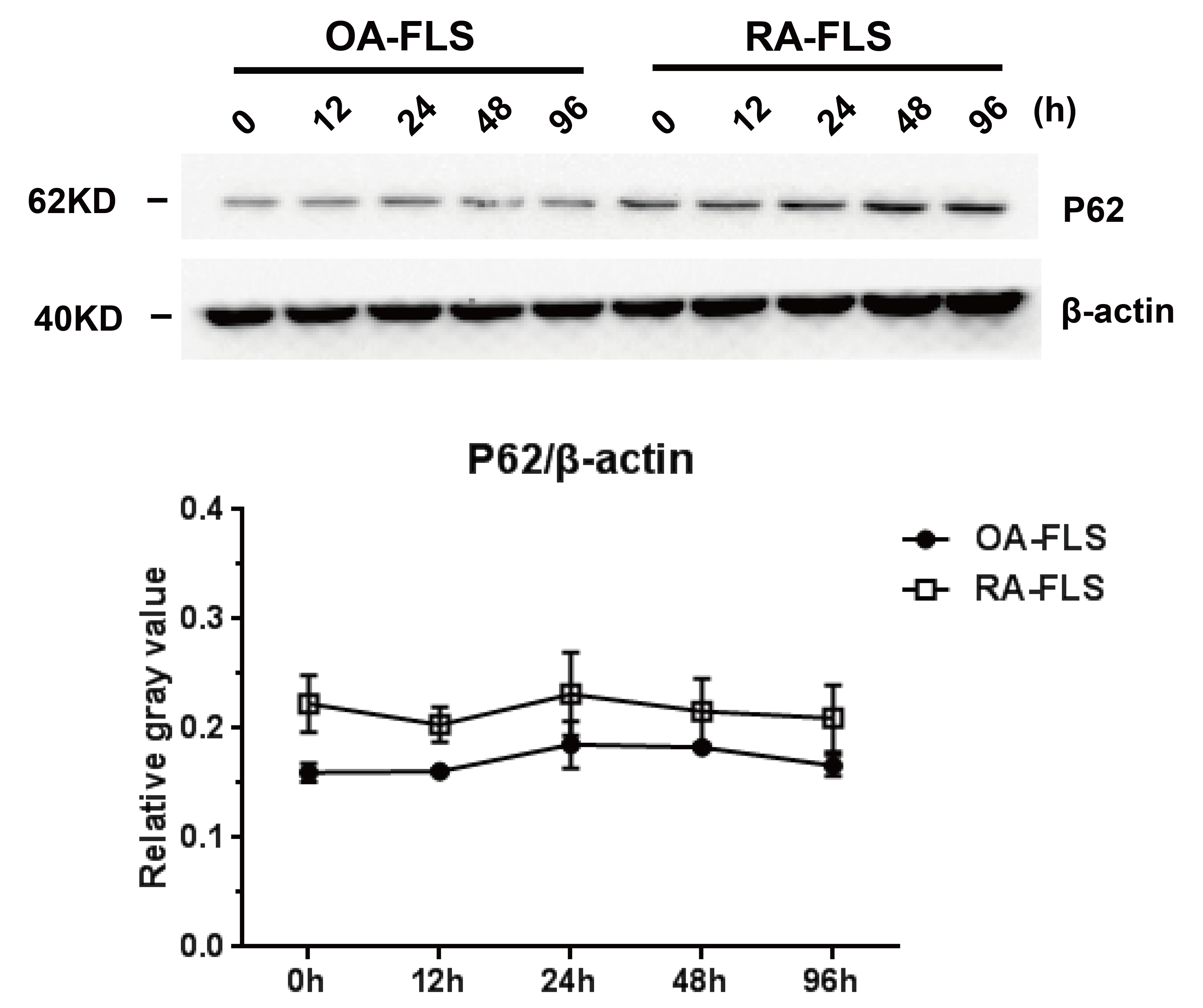

Supplement: Additional file 1: — The expression of P62 in RA-FLS and OA-FLS treated with 0.1μM MTX for the indicated times (TIF 698 kb) [file 13075_2015_892_MOESM1_ESM.tif]
